# 36 Common Data Elements (CDE) in Burn Care Documentation: A Single-center Retrospective Review

**DOI:** 10.1093/jbcr/irac012.039

**Published:** 2022-03-23

**Authors:** Justin Dang, Rendell Bernabe, Joshua Lin, Yuki Kuromaru, Christopher H Pham, Samantha Huang, Megha Sheth, Haig A Yenikomshian, Justin Gillenwater

**Affiliations:** Sidney Kimmel Medical College at Thomas Jefferson University, Costa Mesa, California; LAC + University of Southern California, Los Angeles, California; Keck School of Medicine, University of Southern California, Los Angeles, California; Keck School of Medicine of USC, Los Angeles, California; University of Southern California, Cypress, California; USC, Los Angeles, California; USC Keck School of Medicine, Los Angeles, California; USC/LAC+USC Medical Center, Los Angeles, California; USC/LAC+USC Medical Center, Los Angeles, California

## Abstract

**Introduction:**

Thorough documentation is an important component of delivering quality patient care. Documentation of common data elements (CDE), defined as a precise question with a specified set of responses used across multiple databases or studies, can also assist in improving data collection. Currently, burn care does not have an existing set of CDEs despite their potential to be a reproducible and reliable system for data collection which leads to improved burn care. Our institution performed a retrospective review of patient charts to identify the consistency of our burn care documentation and highlight deficits that could be remedied by the implementation of CDEs.

**Methods:**

This was a single-center retrospective review of patient charts from 2014-2019. Thirty-three CDEs were investigated. Two hundred four patient charts were randomly selected for review. We presented extracted CDEs as frequencies and percentages. Information was obtained from the history and physical notes, progress notes, and discharge summaries.

**Results:**

Our review yielded 204 patient records. The note/record of some data points could not be identified and were excluded from the qualitative calculation. Of the data points that included more than 200 records, 86% percent specified the date of injury, 88% recorded the admission date, 99% reported burn etiology, 94% included total body surface area (TBSA) burned, 94% included burn thickness, 99% specified anatomic injury location, 97% included information about wound care agents/dressings, and 24% recorded the patient’s pain scores. Thirty percent (49/164) reported the presence or absence of inhalation injury. Twenty-six percent (38/148) listed reported presence or absence of non-burn related injuries. Sixty-four percent (127/200) reported presence or absence of comorbid conditions. Other data points were found with varying frequencies (Table 1).

**Conclusions:**

Consistent documentation of burn care remains challenging and many variables are collected inconsistently. Our results highlight the need for CDEs in burn care to standardize documentation.

